# Associations of Serum 25-Hydroxyvitamin D Concentrations and Lipid Profiles Across Adiposity Status Among Children and Adolescents Aged 9–17 Years: A Cross-Sectional Study in Guangzhou, China

**DOI:** 10.3390/nu18132188

**Published:** 2026-07-05

**Authors:** Yujie Peng, Yan Li, Shiyun Luo, Chunzi Zeng, Yuting Qin, Zhifeng Li, Shaofang Song, Guixian Tao, Haonan Li, Jiayi Wan, Zhoubin Zhang, Jie Huang

**Affiliations:** 1 Guangzhou Center for Disease Control and Prevention (Guangzhou Health Supervision Institute), Guangzhou 510440, China; 15103533398@163.com (Y.P.); gzcdcliy@foxmail.com (Y.L.); luoshy25@mail3.sysu.edu.cn (S.L.); chunzi.zeng@163.com (C.Z.); qyt1118@126.com (Y.Q.); jerrphen@icloud.com (Z.L.); anchure@163.com (S.S.); taogx@mail2.sysu.edu.cn (G.T.); lihn26@mail2.sysu.edu.cn (H.L.); wanjy26@mail2.sysu.edu.cn (J.W.); 2 Institute of Public Health, Guangzhou Medical University & Guangzhou Center for Disease Control and Prevention (Guangzhou Health Supervision Institute), Guangzhou 510440, China; 3 School of Public Health, Sun Yat-sen University, Guangzhou 510080, China

**Keywords:** 25-hydroxyvitamin D, dyslipidemia, lipid profile, adiposity, overweight, obesity, children, adolescents

## Abstract

**Background**: Childhood dyslipidemia often tracks into adulthood and contributes to early atherosclerotic changes. Although serum 25-Hydroxyvitamin D (25(OH)D) has been implicated in lipid metabolism, findings in children remain inconsistent, and whether these associations differ by overweight/obesity (ow/ob) status is unclear. We therefore aimed to investigate the associations of serum 25(OH)D concentrations and lipid profiles stratified by ow/ob status among children and adolescents aged 9–17 years. **Methods**: This cross-sectional study included 3067 children and adolescents from Guangzhou, southern China. Anthropometric measurements were obtained by trained staff, and ow/ob status was classified according to WHO criteria. Fasting blood samples were collected to measure serum 25(OH)D and lipid parameters. Multivariable regression analyses were adopted to determine the associations between serum 25(OH)D and lipid profiles. Stratified analysis and interaction tests were further applied according to ow/ob status. **Results**: Higher 25(OH)D concentrations were most consistently associated with lower TG (β = −0.026; 95% CI, −0.040 to −0.011), higher HDL-C (β = 0.025; 95% CI, 0.014 to 0.035), and lower TG/HDL-C ratios (β = −0.044; 95% CI, −0.061 to −0.027). Positive associations were also observed for TC, LDL-C, and non-HDL-C with serum 25(OH)D, but these were attenuated after further adjustment for HDL-C. No significant association was observed for the TC/HDL-C ratio. In stratified analyses, associations with several lipid parameters were observed mainly among children and adolescents without ow/ob, whereas significant inverse associations among those with ow/ob were largely limited to TG and the TG/HDL-C ratio. Significant interactions between vitamin D and ow/ob status were observed for TC and non-HDL-C, but were attenuated after further adjustment for HDL-C. **Conclusions**: In this cross-sectional study, serum 25(OH)D was associated with several lipid parameters among children and adolescents, most consistently with TG, HDL-C, and the TG/HDL-C ratios. Stratified analyses showed different vitamin D–lipid association patterns across ow/ob status, with statistically significant interactions observed only for TC and non-HDL-C. The attenuation of several associations after adjustment for HDL-C suggests that HDL-C may be involved in these observed patterns. Future longitudinal studies are needed to examine causality of vitamin D–lipid associations and the potential role of HDL-C in these associations.

## 1. Introduction

Cardiovascular disease (CVD) continues to be a leading cause of global mortality and morbidity. As a major risk factor for CVD, dyslipidemia originating in childhood often tracks into adulthood and contributes to early atherosclerotic changes [1], underscoring the importance of early prevention. In China, pediatric dyslipidemia has become an increasing public health concern. National surveillance conducted in 2016–2017 reported that approximately 19% of Chinese students aged 6–17 years had at least one abnormal lipid component [2]. A more recent surveillance in Zhejiang Province indicated a significant burden of lipid abnormalities among 6–17-year-old children and adolescents, including elevated TG, TC, LDL-C, and reduced HDL-C [3].

Vitamin D is a fat-soluble hormone traditionally recognized for its role in bone-mineral metabolism, yet suboptimal vitamin D status is highly prevalent in the pediatric population. In China, more than half of children and adolescents have been reported to experience vitamin D insufficiency [4]. Increasing evidence suggested that vitamin D may influence cardiometabolic health beyond skeletal outcomes, including lipid metabolism, although findings remain inconsistent. Observational studies and meta-analyses in the pediatric population have linked lower vitamin D status to more atherogenic lipid profiles, particularly higher TG levels and lower HDL-C concentrations [5,6,7,8,9,10]. Higher serum vitamin D levels have also been associated with lower odds of hypertriglyceridemia, hyper-LDL cholesterolemia, and hypo-HDL cholesterolemia [5,6]. The longitudinal evidence of European pediatric cohorts further suggested that lower baseline 25-hydroxyvitamin D (25(OH)D) levels were associated with adverse TG trajectories independent of adiposity and lifestyle factors [10].

Conversely, associations between vitamin D status and TC or LDL-C have been less consistent, with many studies reporting null findings [5,8,9,10,11,12,13,14]. Evidence for non-HDL-C, an established predictor of atherosclerosis [15], remains limited and inconclusive in pediatric populations [10,12,16,17,18]. Notably, studies in Chinese children and adolescents also suggested regional heterogeneity. For example, vitamin D deficiency was associated with elevated TC and reduced HDL-C in Zhejiang Province [3], whereas no significant associations were observed in Guangzhou [11]. Evidence from supplementation studies further complicates these associations, as meta-analyses of randomized controlled trials (RCTs) have generally failed to demonstrate consistent lipid-lowering effects of vitamin D supplementation, with some reporting modest increases in LDL-C [19,20]. Other studies suggested potential TG benefits only at high cumulative doses, while dose-dependent effects have been observed in cohorts from Denmark and the United States [21,22].

Pediatric overweight and obesity (ow/ob) have risen rapidly globally and in China. National data from the Chinese National Surveys on Students’ Constitution and Health showed that the prevalence of obesity among Chinese children and adolescents aged 7–18 years reached 9.6% in 2019 [23]. The link between pediatric ow/ob and lipid abnormalities has been established; a Chinese community-based study has reported 189% higher odds of dyslipidemia among students with ow/ob than among normal-weight peers [24]. Excess adiposity was also associated with lower circulating concentrations of 25(OH)D concentrations, potentially through volumetric dilution and sequestration of vitamin D in adipose tissue [25]. Meta-analytic evidence reported 41% higher risk of vitamin D deficiency in obese children and adolescents [26].

Emerging evidence indicates that ow/ob may further influence the association between vitamin D status and lipid profiles in pediatric populations. Several studies have discovered significant associations between lower serum 25(OH)D and adverse lipid levels among students with ow/ob [8,10,27,28]. Wolters et al. further suggested that favorable vitamin D–lipid associations were stronger in ow/ob children and adolescents compared to normal-weight counterparts [10]. Some supplementation studies have also reported potential cardiometabolic benefits of vitamin D in pediatric ow/ob populations [29,30]. However, findings remain inconsistent, with a Chinese cross-sectional study showing no significant differences in vitamin D–dyslipidemia associations by obesity status [31], and meta-analyses of RCTs providing limited support for the lipid benefits of vitamin D supplementation, particularly at lower doses [32,33].

Taken together, the associations between vitamin D status and lipid profiles in children and adolescents remain controversial, especially for Chinese populations. Direct comparisons of vitamin D–lipid associations across different weight statuses are also limited. Therefore, our present study aimed to explore whether the associations between serum 25(OH)D and lipid profiles differed by overweight/obesity status among children and adolescents in Guangzhou, southern China. We hypothesized that higher 25(OH)D levels would be associated with favorable lipid profiles and that these associations would differ by weight status. This study may provide evidence relevant to early CVD risk prevention in southern Chinese youth.

## 2. Materials and Methods

### 2.1. Participants

This cross-sectional study, conducted from March 2023 to May 2024, applied a multistage, stratified cluster random sampling strategy to recruit children and adolescents aged 9–17 years in Guangzhou, China. First, five primary schools, five middle schools, four high schools, and one all-grade school were randomly selected from four urban and rural districts of Guangzhou. Second, six grades were selected via stratified random sampling, including three grades from primary schools, two grades from middle schools, and one grade from high schools. Third, two to four classes were randomly sampled from each grade.

The required sample size was calculated using the following formula: $N=deff\frac{{µ}_{\alpha /2}^{2}P\left(1-P\right)}{\delta^{2}}$. A two-sided confidence level of 95% was applied (µ_α/2_ = 1.96). The probability (P) of vitamin D deficiency was set as 19%, according to national estimates among Chinese children aged 6–17 years [34]. The design effect (deff) was specified as 3, and the allowable error (δ) was defined as 15% of P. Based on these parameters, the minimal sample size was calculated to be 2184 participants. To compensate for invalid or missing data, the target sample size was increased by 10%, yielding a final required sample of at least 2402 participants. The participant selection process is shown in Figure S1. Ultimately, a total of 3067 school-aged children were included in the analysis after excluding individuals without basic information, anthropometric and laboratory data, or supplemental information. This study was in accordance with the Declaration of Helsinki and was approved by the Ethics Committee of the Guangzhou Center for Disease Control and Prevention (approval numbers: GZCDC-ECHR-2022P0036). Written informed consent was obtained from all participants and their legal guardians prior to study enrollment.

### 2.2. Data Collection

Sociodemographic, family history, and lifestyle information—including age, sex, grade, residential location, parental history of dyslipidemia, outdoor activity, smoking, drinking, and vitamin D supplementation—was collected by a self-reported questionnaire. A semi-quantitative food frequency questionnaire (FFQ) was used to assess children’s dietary intake over the previous month, capturing both consumption frequency and portion size with the aid of food photographs and models. The self-reported questionnaire and FFQ were adapted from our previous study [34]. Intake of saturated fat and cholesterol was calculated from FFQ data.

### 2.3. Anthropometric and Laboratory Measurements

Anthropometric measurements were all performed by trained physicians adhering to the standard of Human Health Monitoring Anthropometric Methods (WS/T424-2013) [35]. Height was measured to the nearest 0.1 cm using a metallic column stadiometer, and weight was measured to the nearest 0.1 kg using an electronic scale. Waist circumference was measured to the nearest 0.1 cm with fiberglass tape, and body mass index (BMI) was calculated as weight (kg)/height (m)^2^; BMI-for-age z-scores (BMI z-score) were derived using the 2007 World Health Organization growth reference standards [36], while the waist-to-height ratio (WHtR) was calculated as waist circumference (cm)/height (cm). Sitting blood pressure was measured twice at 1 min intervals following a 5 min rest using a validated mercury sphygmomanometer, with cuff size selected according to mid-upper arm circumference; a third measurement was requested if the difference between previous two measurements was more than 10 mmHg. The mean of the two closest measurements was used for analysis.

Following an overnight fast, venous blood samples were collected from all participants to assess serum 25(OH)D, fasting blood glucose (FBG), TG, TC, HDL-C, LDL-C, and non-HDL-C. Serum 25(OH)D concentrations were measured using the Waters Acqui (liquid phase) liquid chromatography and Xevo TQ-S mass spectrometry systems [34].

### 2.4. Definitions

Although there is no consensus on the definition of vitamin D status, we applied a more widely recommended standard for generally healthy populations from the Institute of Medicine (IOM) [37]. Serum 25(OH)D concentrations <12 ng/mL, 12 to <20 ng/mL, and ≥20 ng/mL were classified as vitamin D deficiency, inadequacy, and adequacy, respectively. Vitamin D insufficiency was defined as the combination of deficiency and inadequacy.

Cardiovascular risk factors were defined according to international and Chinese expert consensus statements for children and adolescents, specifically the following:

(1) Overweight or obesity (OW/OB) was defined as a BMI z-score > 1 [38];
(2) Central obesity was defined as a waist circumference ≥ 90th percentile for age and sex [39];
(3) Elevated TG was defined as ≥1.1 mmol/L for children <10 years or ≥1.5 mmol/L for those ≥10 years [40];
(4) Elevated TC was defined as ≥5.2 mmol/L [40];
(5) Elevated LDL-C was defined as ≥3.4 mmol/L [40];
(6) Reduced HDL-C is defined as <1.0 mmol/L [40];
(7) Elevated non-HDL-C was defined as ≥3.7 mmol/L [40];
(8) Total dyslipidemia was defined as the presence of at least one abnormal lipid parameter (TG, TC, LDL-C, or HDL-C) [40];
(9) Elevated fasting glucose was defined as ≥5.6 mmol/L [41];
(10) Elevated blood pressure was defined as systolic or diastolic blood pressure ≥ 90th percentile for age, sex, and height [42].

The dietary diversity score (DDS) was calculated using nine major food groups: cereals; white root and tuber crops; legumes, legume products, nuts and seeds; vegetables; fruits; meat and poultry; eggs; fish and seafood; and milk and dairy foods. A total DDS ranging from one to nine points was calculated by awarding one point for the consumption of each food group in the previous month [43]. Dietary diversity status was categorized as high or low according to the median dietary diversity score (DDS).

### 2.5. Statistical Analysis

Data is presented as mean (SD) for normally distributed variables, median (IQR) for skewed variables, and frequency (%) for categorical variables. Differences in participant characteristics across serum 25(OH)D categories were compared using the χ^2^ test for categorical variables, one-way analysis of variance for normally distributed continuous variables, and the Kruskal–Wallis test for skewed variables.

In the multivariable analysis, selection of confounders was based on biological relevance and the prior literature. Covariates included age, sex, season of blood collection, residential location, socioeconomic indicators, lifestyle factors, dietary factors, anthropometric measures, and other cardiometabolic indicators. Skewed variables were log-transformed prior to inclusion in regression models.

Serum 25(OH)D was modeled as a continuous variable standardized to one standard deviation (SD), and β coefficients were interpreted as the change in lipid associated with each SD increase in serum 25(OH)D. In addition, the β coefficients of lipid parameters across different vitamin D status categories were calculated using a general linear model. Then, the trend was tested by assigning vitamin D status as a continuous variable. Multivariable logistic regression models were applied to examine associations between vitamin D status and lipid abnormalities, with adjusted odds ratios (ORs) and 95% confidence intervals (CIs) calculated per the standard deviation increase in serum 25(OH)D.

Restricted cubic spline functions with four knots were fitted within generalized additive models to assess potential non-linear associations between vitamin D and lipid parameters. Overall associations and departures from linearity were evaluated using Wald tests. Adjusted dose–response curves with 95% CIs were plotted, using clinical reference values for lipid concentrations and mean values for lipid ratios. Threshold effects were further examined using segmented linear regression models, which estimated inflection points and segment-specific slopes for the 25(OH)D–HDL-C association and compared model fit with linear models using likelihood ratio tests.

We further tested for effect modification on both multiplicative and additive scales. For continuous lipid outcomes, multivariable linear regression models included a 25(OH)D × ow/ob interaction term, and interaction plots were generated to show predicted non-HDL-C and TC across 25(OH)D level stratified by weight status. For HDL-C, due to non-linear association with vitamin D, the interaction was assessed using a likelihood ratio test comparing models with and without the interaction term. Forest plots were constructed to visually display ow/ob-stratified associations between 25(OH)D and lipid outcomes, presenting multivariable-adjusted effect estimates and 95% confidence intervals for each subgroup. For dichotomous dyslipidemia outcomes, interaction terms were included in logistic regression models, and additive interaction was assessed using the relative excess risk due to interaction (RERI), with 95% CIs including zero indicating the absence of an additive interaction. Explanatory analysis was conducted by adjusting for additional HDL-C. Two sensitivity analyses were performed to verify the robustness of our results. First, participants with a BMI z-score < −2 were excluded. Second, we further stratified ow/ob into ow (1 < BMI z-score ≤ 2) and ob (BMI z-score > 2) subgroups according to WHO criteria [39]. All statistical analyses were performed at R 4.5.0. All tests were two-sided and were considered statistically significant when *p* value < 0.05.

## 3. Results

### 3.1. Characteristics of Participants

A total of 3067 children and adolescents (53.41% boys) were included in the analysis with a median age of 13.67 years. As shown in Table 1, the overall prevalence of vitamin D insufficiency was 61.59% (12.49% for deficiency), and insufficient serum 25(OH)D was significantly associated with older participants, girls, and those living in rural areas (all *p* < 0.001). Children and adolescents with adequate vitamin D status more frequently reported vitamin D supplementation, longer duration of outdoor activity, higher dietary diversity, and lower frequency of SSB consumption (all *p* < 0.05). BMI z-score differed modestly across vitamin D categories, whereas WHtR did not.

Lipid profiles also varied according to vitamin D status. Participants with adequate vitamin D had notably lower TG, higher HDL-C, and lower TG/HDL-C ratios compared with those with vitamin D deficiency (TG: 0.81 vs. 0.84 mmol/L; HDL-C: 1.38 vs. 1.31 mmol/L; TG/HDL-C ratio: 0.59 vs. 0.65; all *p* < 0.05). The prevalence of reduced HDL-C was highest among vitamin D-deficient children and lowest among those with adequate levels (12.79% vs. 6.96%, *p* < 0.001), while the prevalence of other cardiometabolic abnormalities did not differ significantly across vitamin D status.

### 3.2. Associations Between Serum 25(OH)D and Lipid Profiles

In fully adjusted models, higher serum 25(OH)D levels were associated with several lipid parameters (Table 2). Each 1-SD increase in serum 25(OH)D was significantly associated with higher HDL-C (β = 0.025; 95% CI, 0.014 to 0.035), lower TG (β = −0.026; 95% CI, −0.040 to −0.011), and lower TG/HDL-C ratios (β = −0.044; 95% CI, −0.061 to −0.027). Positive associations were also observed for TC (β = 0.057; 95% CI, 0.027 to 0.087), LDL-C (β = 0.021; 95% CI, 0.000 to 0.042), and non-HDL-C (β = 0.032; 95% CI, 0.007 to 0.057), whereas no significant association was observed for the TC/HDL-C ratios. When the lipid outcome was categorized, higher serum 25(OH)D levels were only associated with higher odds of elevated TC (OR = 1.22; 95% CI, 1.03 to 1.43) in fully adjusted models (Table S1). No statistically significant associations were found for elevated TG, elevated LDL-C, reduced HDL-C, elevated non-HDL-C, or overall dyslipidemia.

**Figure 1:**
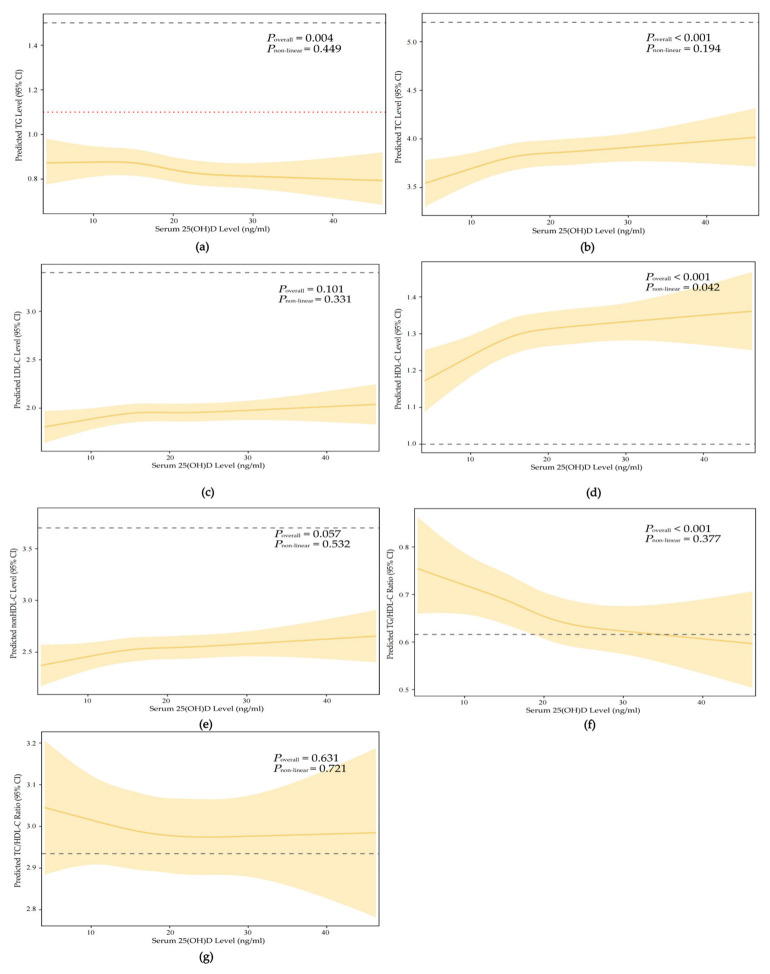
Restricted cubic spline analyses of the associations between serum 25(OH)D levels and lipid profiles. (**a**) TG; (**b**) TC; (**c**) LDL-C; (**d**) HDL-C; (**e**) non-HDL-C; (**f**) TG/HDL-C ratio; and (**g**) TC/HDL-C ratio. Associations were adjusted for the full model. Black dashed lines indicate clinical cut-offs of lipid parameters (TG, TC, LDL-C, HDL-C, non-HDL-C) and means for lipid ratios (TG/HDL-C, TC/HDL-C); red dotted lines indicate age-specific clinical cut-offs for participants aged <10 years; solid yellow lines indicate predicted lipid levels; shaded yellow areas indicate 95% CI.

Categorical analyses showed consistent dose–response patterns (Table 2). Compared with participants with vitamin D deficiency, those with sufficient vitamin D had higher TC, LDL-C and HDL-C, as well as lower TG and TG/HDL-C ratios (all *p* for trend < 0.05). Further RCS analysis (Figure 1) demonstrated a significant non-linear association between serum 25(OH)D and HDL-C (*p* for non-linearity = 0.042), characterized by a steeper increase at lower and a plateau at higher 25(OH)D concentrations (Table S2). Associations with atherogenic lipids, TG/HDL-C, and TC/HDL-C ratios were monotonic or null, with no evidence of non-linearity (Figure 1).

### 3.3. Interaction Between Serum 25(OH)D and ow/ob Status on Lipid Profiles

**Figure 2:**
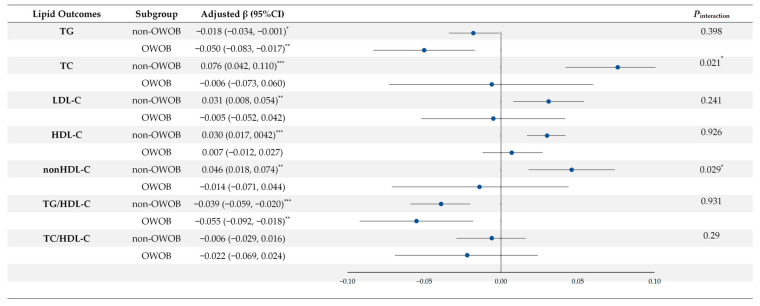
Associations between serum 25(OH)D and lipid profiles stratified by ow/ob status. Adjusted for the full model except BMI z-score. * *p* < 0.05, ** *p* < 0.01, *** *p* < 0.001.

**Figure 3:**
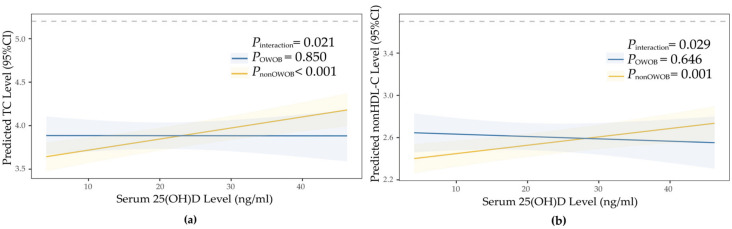
Interaction plots of serum 25(OH)D levels and ow/ob status on non-HDL-C and TC levels. (**a**) TC and (**b**) non-HDL-C. Associations were adjusted for the full model. Gray dashed lines indicate clinical cut-offs of lipid profiles.

Figure 2 and Table S3 demonstrate the associations between vitamin D and lipid profiles stratified by ow/ob status after adjusting for confounders. Among children and adolescents without ow/ob, each 1-SD increase in serum 25(OH)D was significantly associated with lower TG (β = −0.018; 95% CI, −0.034 to −0.001), higher TC (β = 0.076; 95% CI, 0.042 to 0.110), higher LDL-C (β = 0.031; 95% CI, 0.008 to 0.054), higher HDL-C (β = 0.030; 95% CI, 0.017 to 0.042), higher non-HDL-C (β = 0.046; 95% CI, 0.018 to 0.074), and lower TG/HDL-C ratios (β = −0.039; 95% CI, −0.059 to −0.020). In contrast, significant associations were only notable for TG and TG/HDL-C ratios among ow/ob children and adolescents. Associations with other atherogenic lipid parameters in the ow/ob group did not reach statistical significance. In addition, significant multiplicative interactions between vitamin D level and ow/ob status were exclusively observed for TC (*p* for interaction = 0.021) and non-HDL-C (*p* for interaction = 0.029) (Figure 3). When serum 25(OH)D was modeled categorically, significant trends were found in TC, LDL-C, HDL-C, non-HDL-C, and TG/HDL-C ratios among children and adolescents without ow/ob (all *p* for trend < 0.05), but these trends were not evident in the ow/ob group (Table S3).

For lipid abnormalities, higher serum 25(OH)D tended to be associated with 25% lower odds of reduced HDL-C (OR = 0.75; 95% CI, 0.62 to 0.91) and 25% higher odds of elevated TC (OR = 1.25; 95% CI, 1.03 to 1.52) for non-ow/ob children and adolescents (Table S4). Nevertheless, none of the associations between vitamin D and lipid abnormalities were notable in ow/ob participants, except for elevated TG. No statistically significant multiplicative or additive interactions between serum 25(OH)D levels and ow/ob status were observed for binary lipid outcomes (Table S4).

### 3.4. Explanatory and Sensitivity Analyses

To further explore whether HDL-C contributed to the observed associations between vitamin D and lipid parameters, additional models were fitted with HDL-C as an additional covariate. After further adjustment for HDL-C, the inverse association between serum 25(OH)D levels and TG persisted in all participants (β = −0.024; 95% CI, −0.039 to −0.010), whereas the associations with other atherogenic lipids were no longer statistically significant (Table S5). Additionally, the significant interaction between vitamin D and ow/ob status was attenuated after further adjustment for HDL-C. In stratified analyses, the associations between vitamin D and lipids were no longer evident in the non-ow/ob group, but the inverse association with TG remained among those with ow/ob.

Two sensitivity analyses were conducted to examine the robustness of our results (Tables S6 and S7). The associations of serum 25(OH)D with lipid measures largely remained consistent under each assumption. The significant interaction of vitamin D and ow/ob status also persisted for TC and non-HDL-C in two analyses. However, the non-linear association between vitamin D and HDL-C was no longer significant (*p* = 0.089) after excluding thin participants.

## 4. Discussion

In this large cross-sectional study, vitamin D insufficiency was highly prevalent, with more than half of participants classified as having insufficient vitamin D status. Higher serum 25(OH)D concentrations were most consistently associated with lower TG, higher HDL-C, and lower TG/HDL-C ratios, while the associations with TC, LDL-C, and non-HDL-C were positive and no significant association was observed for the TC/HDL-C ratio. In addition, a non-linear association was exhibited between vitamin D and HDL-C, with a marked increase from vitamin D deficiency to inadequacy and diminishing gains at higher 25(OH)D concentrations. Weight-stratified analyses demonstrated that vitamin D–lipid associations differed by ow/ob status, although statistically significant interactions were limited to TC and non-HDL-C.

Our observations regarding TG, HDL-C, and TG/HDL-C ratios align with prior pediatric studies reporting more favorable changes at higher 25(OH)D levels [5,6,8,9,10,16]. Notably, the inverse relationship between vitamin D and TG remained evident after additional adjustment for HDL-C (Table S5), supporting the relative robustness of the TG finding across different models. Experimental studies have proposed several biological pathways that may link vitamin D to TG metabolism, including inhibition of sterol regulatory element-binding protein (SREBP) activation via enhanced degradation of SREBP cleavage-activating protein and increased lipoprotein lipase activity [44,45]. These mechanisms may reduce TG production and enhance lipolysis. The TG/HDL-C ratio is widely used as an index of the atherogenic pathway and insulin resistance [46], and its inverse association with 25(OH)D may partly reflect abnormal glucose metabolism that was not fully captured by fasting glucose alone; however, we did not directly measure insulin resistance-related parameters. It is worth noting that the effect size of vitamin D–lipid associations was modest, which may explain the absence of significant associations for most dichotomous dyslipidemia outcomes (Table S1).

Unexpectedly, we observed positive associations between serum 25(OH)D with TC, LDL-C, and non-HDL-C. Although many pediatric studies have reported null associations for these lipid fractions, similar positive correlations have been documented in female adolescents in Saudi Arabia [7]. Several findings from our study suggested that these positive associations may not reflect independent relationships of vitamin D with apoB-containing lipoproteins. First, these associations were substantially attenuated after additional adjustment for HDL-C (Table S5), suggesting that they may reflect concurrent HDL-linked changes rather than selective associations with atherogenic lipid fractions. Second, RCS analyses showed no statistically significant overall associations between vitamin D and LDL-C or non-HDL-C, supporting limited robustness for these associations. Third, the absence of an association with the TC/HDL-C ratio implies that TC and HDL-C may vary in parallel, which is more compatible with compositional lipid changes than with a direct unfavorable association with apoB-containing lipids. The potential explanatory role of HDL-C in vitamin D–lipid associations may be explained by cholesteryl ester transfer protein (CETP)-mediated cholesterol exchange between HDL and apoB-containing lipoproteins [47].

When vitamin D status was categorized, dose–response trends were exhibited for most lipid parameters. Specifically, the association with HDL-C showed a threshold-like pattern, with a more pronounced increase in HDL-C occurring from vitamin D deficiency to inadequacy and smaller gains at higher 25(OH)D concentrations. This finding partly aligns with previous meta-analysis reporting non-linear associations between vitamin D status and lipid profiles [6], although the vitamin D ranges at which HDL-C changes are most evident may vary across studies. In sensitivity analysis, the non-linear association between 25(OH)D and HDL-C levels was attenuated after excluding thin children and adolescents, implying that thin children and adolescents with lower vitamin D status may have contributed to greater variability in HDL-C concentrations.

The prevalence of ow/ob in our pediatric participants was 21.65%, slightly lower than estimates from earlier national surveys [48]. This difference may reflect regional differences in lifestyle and dietary patterns. In weight-stratified analyses, the associations between vitamin D and lipid parameters in the non-ow/ob group resembled those observed in the overall population. In contrast, among participants with ow/ob, statistically significant inverse associations were mainly confined to TG and TG/HDL-C ratios, while associations with other lipid parameters did not reach statistical significance.

Several biological mechanisms may partly explain why excess adiposity could influence vitamin D–lipid associations. In individuals with excess fat, expanded volume of adipose tissue may contribute to sequestration and volumetric dilution of vitamin D, resulting in lower circulating 25(OH)D levels [49,50]. Impaired liver function related to excess fat may also blunt the formation of vitamin D [51]. Additionally, visceral adipose tissue secretes pro-inflammatory cytokines, such as IL-6 and TNF-α, which may further impair vitamin D signaling by downregulating the expression of the vitamin D receptor (VDR) and are often accompanied by dyslipidemia [49,50]. Moreover, insulin resistance associated with both obesity and vitamin D deficiency may contribute to abnormal lipid metabolism [51]. Consistent with these plausible mechanisms, evidence of an interaction between vitamin D and ow/ob status was observed in our study, but only limited to TC and non-HDL-C. Notably, these interactions were attenuated in models additionally adjusting for HDL-C (Table S5), implying HDL-linked pathways may be related to the observed differences in vitamin D–lipid associations by ow/ob status.

Our study has several strengths. First, we directly compared vitamin D–lipid associations between ow/ob and non-ow/ob children and adolescents, strengthening evidence on adiposity-related heterogeneity. Second, the large and well-characterized sample provided sufficient statistical power to detect modest associations. Third, the additional adjustment for HDL-C allows for the exploration of its potential role in vitamin D–lipid associations. However, some limitations should be acknowledged. First, Tanner stage and sex hormone levels were not assessed; the lack of pubertal indicators may have introduced residual confounding. Second, outdoor activity was assessed by self-report and may not comprehensively reflect physical activity patterns. Third, the generalizability of our findings to all Chinese children and adolescents may be limited due to the region-specific nature of our study. Finally, owing to the cross-sectional design, our study cannot establish a causal inference between vitamin D status and lipid profiles. Future longitudinal studies are warranted to examine the causal relationship between vitamin D and lipid profiles and to evaluate their implications for cardiovascular risk during childhood and adolescence.

## 5. Conclusions

In conclusion, higher serum 25-hydroxyvitamin D concentrations were most consistently associated with lower TG, higher HDL-C, and a lower TG/HDL-C ratio among children and adolescents aged 9–17 years in Guangzhou. Stratified analyses showed different vitamin D–lipid association patterns across overweight and obesity status, with statistically significant interactions observed only for TC and non-HDL-C. The positive associations of serum 25(OH)D with TC, LDL-C, and non-HDL-C, as well as the observed interactions with overweight/obesity status, were attenuated after additional adjustment for HDL-C, suggesting that HDL-C may be involved in these observed patterns. Further longitudinal studies are needed to examine the causal relationships between vitamin D status and lipid profiles and to investigate the potential role of HDL-C in these associations.

## Figures and Tables

**Table 1:** Participants’ characteristics, stratified by serum 25(OH)D status.

Characteristics	Total (*n* = 3067)	Deficiency (*n* = 383)	Inadequacy (*n* = 1506)	Adequacy (*n* = 1178)	*p*-Value
**Sociodemographics & Family History**					
Age groups (*n*(%))					<0.001 ***
9–11 years	900 (29.34)	81 (21.15)	445 (29.55)	374 (31.75)	
12–14 years	1132 (36.91)	134 (34.99)	492 (32.67)	506 (42.95)	
15–17 years	1035 (33.75)	168 (43.86)	569 (37.78)	298 (25.30)	
Gender (*n*(%))					<0.001 ***
Boys	1638 (53.41)	115 (30.03)	756 (50.20)	767 (65.11)	
Girls	1429 (46.59)	268 (69.97)	750 (49.80)	411 (34.89)	
Education (*n*(%))					<0.001 ***
Primary School	1015 (33.09)	90 (23.50)	496 (32.93)	429 (36.42)	
Middle School	1035 (33.75)	129 (33.68)	445 (29.55)	461 (39.13)	
High School	1017 (33.16)	164 (42.82)	565 (37.52)	288 (24.45)	
Location (*n*(%))					<0.001 ***
Urban	1613 (52.59)	96 (25.07)	736 (48.87)	781 (66.30)	
Rural	1454 (47.41)	287 (74.93)	770 (51.13)	397 (33.70)	
Month of blood collection (*n*(%))					0.002 **
Spring	2698 (87.97)	329 (85.90)	1302 (86.45)	1067 (90.58)	
Fall	369 (12.03)	54 (14.10)	204 (13.55)	111 (9.42)	
Parental history of dyslipidemia (*n*(%))	80 (2.61)	9 (2.35)	37 (2.46)	34 (2.89)	0.743
**Anthropometrics**					
BMI z-score (mean (SD))	0.01 (1.28)	−0.11 (1.25)	−0.01 (1.30)	0.07 (1.27)	0.036 *
WHtR (mean (SD))	0.41 (0.05)	0.41 (0.05)	0.41 (0.05)	0.42 (0.05)	0.299
**Lifestyle & Diet**					
Outdoor activity time, hrs/d (median [IQR])	1.50 [0.92, 2.50]	1.25 [0.75, 2.00]	1.50 [0.83, 2.50]	1.75 [1.00, 2.75]	<0.001 ***
Vitamin D supplement use (*n*(%))	345 (11.25)	25 (6.53)	156 (10.36)	164 (13.92)	<0.001 ***
Smoking (*n*(%))	117 (3.81)	14 (3.66)	46 (3.05)	57 (4.84)	0.056
Drinking (*n*(%))	543 (17.70)	51 (13.32)	260 (17.26)	232 (19.69)	0.015 *
Dietary diversity (*n*(%))					0.005 **
High-level DDS	2100 (68.47)	239 (62.40)	1023 (67.93)	838 (71.14)	
Low-level DDS	967 (31.53)	144 (37.60)	483 (32.07)	340 (28.86)	
Saturated fat intake, g/d (median [IQR])	12.54 [8.73, 18.14]	11.97 [8.33, 17.28]	12.21 [8.51, 17.62]	13.18 [9.21, 18.90]	<0.001 ***
Cholesterol intake, mg/d (median [IQR])	335.44 [211.03, 510.89]	270.08 [185.31, 423.25]	323.00 [204.53, 484.49]	383.68 [238.91, 568.63]	<0.001 ***
Frequency of SSB ≥ once/wk (*n*(%))	2265 (73.85)	305 (79.63)	1102 (73.17)	858 (72.84)	0.022 *
**Lipid profiles**					
TG, mmol/L (median [IQR])	0.82 [0.64, 1.10]	0.84 [0.64, 1.14]	0.83 [0.67, 1.11]	0.81 [0.63, 1.06]	0.014 *
TC, mmol/L (mean (SD))	3.94 (0.78)	3.77 (0.77)	3.94 (0.78)	4.00 (0.79)	<0.001 ***
LDL-C, mmol/L (mean (SD))	2.01 (0.55)	1.90 (0.53)	2.01 (0.55)	2.04 (0.55)	<0.001 ***
HDL-C, mmol/L (mean (SD))	1.37 (0.28)	1.31 (0.26)	1.37 (0.28)	1.38 (0.28)	<0.001 ***
Non-HDL-C, mmol/L (mean (SD))	2.58 (0.66)	2.46 (0.66)	2.57 (0.66)	2.62 (0.66)	<0.001 ***
TG/HDL-C (median [IQR])	0.62 [0.46, 0.85]	0.65 [0.48, 0.90]	0.63 [0.47, 0.86]	0.59 [0.45, 0.81]	<0.001 ***
TC/HDL-C (mean (SD))	2.93 (0.54)	2.93 (0.58)	2.93 (0.54)	2.94 (0.53)	0.770
**Cardiometabolic Abnormalities**					
Elevated TG (*n*(%))	276 (9.00)	43 (11.23)	133 (8.83)	100 (8.49)	0.253
Elevated TC (*n*(%))	176 (5.74)	14 (3.66)	84 (5.58)	78 (6.62)	0.089
Elevated LDL-C (*n*(%))	48 (1.57)	3 (0.78)	23 (1.53)	22 (1.87)	0.327
Reduced HDL-C (*n*(%))	238 (7.76)	49 (12.79)	107 (7.10)	82 (6.96)	<0.001 ***
Elevated non-HDL-C (*n*(%))	169 (5.51)	14 (3.66)	86 (5.71)	69 (5.86)	0.232
Total dyslipidemia (*n*(%))	631 (20.57)	93 (24.28)	298 (19.79)	240 (20.37)	0.148
Elevated fasting glucose (*n*(%))	152 (4.96)	16 (4.18)	69 (4.58)	67 (5.69)	0.320
Elevated blood pressure (*n*(%))	293 (9.55)	34 (8.88)	140 (9.30)	119 (10.10)	0.695
Ow/ob (*n*(%))					0.117
Yes	664 (21.65)	70 (18.28)	321 (21.31)	273 (23.17)	
No	2403 (78.35)	313 (81.72)	1185 (78.69)	905 (76.83)	
Central-obese (*n*(%))	371 (12.10)	46 (12.01)	184 (12.22)	141 (11.97)	0.980

Note: Data is presented as mean (SD) for normally distributed variables, median [IQR] for skewed variables, and *n* (%) for categorical variables. * *p* < 0.05, ** *p* < 0.01, *** *p* < 0.001.

**Table 2:** Associations between serum 25(OH)D concentrations and lipid profiles.

Lipid Profiles	VD Change/Status	Model 1		Model 2		Model 3	
		β (95%) CI	*p* for Trend	β (95%) CI	*p* for Trend	β (95%) CI	*p* for Trend
TG ^#^	Per SD	−0.024 (−0.037, −0.010) ***	/	−0.024 (−0.039, −0.009) **	/	−0.026 (−0.040, −0.011) ***	/
	Deficiency	Reference	0.010 *	Reference	0.022 *	Reference	0.028 *
	Insufficient	−0.008 (−0.051, 0.036)		−0.016 (−0.060, 0.028)		−0.015 (−0.059, 0.028)	
	Sufficient	−0.045 (−0.090, −0.001) *		−0.016 (−0.060, 0.028)		−0.045 (−0.092, 0.002)	
TC	Per SD	0.069 (0.041, 0.097) ***	/	0.063 (0.033, 0.093) ***		0.057 (0.027, 0.087) ***	/
	Deficiency	Reference	<0.001 ***	Reference	<0.001 ***	Reference	<0.001 ***
	Inadequate	0.175 (0.087, 0.262) ***		0.143 (0.056, 0.231) **		0.125 (0.037, 0.213) **	
	Adequate	0.236 (0.146, 0.326) ***		0.207 (0.112, 0.302) ***		0.185 (0.089, 0.281) ***	
LDL-C	Per SD	0.039 (0.020, 0.059) ***	/	0.025 (0.004, 0.045) *	/	0.021 (0.000, 0.042) *	/
	Deficiency	Reference	<0.001 ***	Reference	0.021 *	Reference	0.035 *
	Inadequate	0.111 (0.050, 0.173) ***		0.073 (0.011, 0.134) *		0.065 (0.004, 0.126) *	
	Adequate	0.140 (0.077, 0.204) ***		0.089 (0.023, 0.156) **		0.081 (0.015, 0.148) *	
HDL-C	Per SD	0.021 (0.011, 0.030) ***	/	0.025 (0.014, 0.035) ***	/	0.025 (0.014, 0.035) ***	/
	Deficiency	Reference	<0.001 ***	Reference	<0.001 ***	Reference	<0.001 ***
	Inadequate	0.060 (0.029, 0.091) ***		0.064 (0.033, 0.096) ***		0.060 (0.029, 0.091) ***	
	Adequate	0.075 (0.043, 0.107) ***		0.086 (0.052, 0.120) ***		0.082 (0.048, 0.116) ***	
Non-HDL-C	Per SD	0.048 (0.025, 0.072) ***	/	0.038 (0.013, 0.063) **	/	0.032 (0.007, 0.057) *	/
	Deficiency	Reference	<0.001 ***	Reference	0.004 **	Reference	0.013 *
	Inadequate	0.114 (0.040, 0.188) **		0.079 (0.005, 0.153) *		0.065 (−0.009, 0.139)	
	Adequate	0.160 (0.084, 0.237) ***		0.121 (0.041, 0.201) **		0.103 (0.023, 0.184) *	
TG/HDL-C ^#^	Per SD	−0.039 (−0.055, −0.023) ***	/	−0.042 (−0.060, −0.025) ***	/	−0.044 (−0.061, −0.027) ***	/
	Deficiency	Reference	<0.001 ***	Reference	<0.001 ***	Reference	<0.001 ***
	Inadequate	−0.053 (−0.104, −0.003) *		−0.064 (−0.116, −0.012) *		−0.060 (−0.109, −0.010) *	
	Adequate	−0.102 (−0.154, −0.050) ***		−0.064 (−0.116, −0.012) ***		−0.105 (−0.159, −0.051) ***	
TC/HDL-C	Per SD	0.007 (−0.012, 0.026)	/	−0.007 (−0.028, 0.014)	/	−0.011 (−0.031, 0.010)	/
	Deficiency	Reference	0.523	Reference	0.453	Reference	0.391
	Inadequate	0.000 (−0.061, 0.061)		−0.036 (−0.098, 0.026)		−0.037 (−0.096, 0.023)	
	Adequate	0.015 (−0.048, 0.077)		−0.036 (−0.098, 0.026)		−0.037 (−0.102, 0.028)	

Note: Model 1: crude model; Model 2: adjusted for sex, age, education, location, and month of blood collection; Model 3: further adjusted for family history of dyslipidemia, BMI z-scores, WHtR, outdoor activity time, vitamin D supplement use, smoking, drinking, DDS, frequency of SSB over once/wk, and intake of saturated fat and cholesterol. ^#^ Outcomes were log-transformed due to their skewness; exponentiated β represents multiplicative changes in the outcome. “/” denotes data not applicable. * *p* < 0.05, ** *p* < 0.01, *** *p* < 0.001.

## Data Availability

The datasets used and analyzed during the current study are available from the corresponding author upon reasonable request due to privacy.

## Supplementary Materials

The following supporting information can be downloaded at: https://www.mdpi.com/article/10.3390/nu18132188/s1, Figure S1: Flowchart of participant selection; Table S1: Associations between serum 25(OH)D change and dyslipidemia outcomes; Table S2: The threshold effect of serum 25(OH)D levels on HDL-C; Table S3: Associations of serum 25(OH)D status and lipid profiles, stratified by ow/ob status; Table S4: Interaction of serum 25(OH)D levels and ow/ob on dyslipidemia outcomes; Table S5: Interaction of serum 25(OH)D level and ow/ob status on lipid profiles after adjusting the HDL-C; Table S6: Interaction of serum 25(OH)D level and ow/ob status on lipid profiles after excluding children and adolescents with thinness; Table S7: Interaction of serum 25(OH)D level and weight status on lipid profiles.

## Abbreviations

The following abbreviations are used in this manuscript.

25(OH)D	25-hydroxyvitamin D
ow/ob	Overweight or obesity
ow	Overweight
ob	Obesity
WHtR	Waist-to-height ratio
BMI	Body mass index
DDS	Dietary diversity scores
SSB	Sugar sweetened beverages
TG	Triglycerides
TC	Total cholesterol
LDL-C	Low-density lipoprotein cholesterol
HDL-C	High-density lipoprotein cholesterol
Non-HDL-C	Non-high-density lipoprotein cholesterol
OR	Odds ratio
CI	Confidence interval
RERI	Relative excess risk due to interaction
