# Supplementary material for: Associations of Serum 25-Hydroxyvitamin D Concentrations and Lipid Profiles Across Adiposity Status Among Children and Adolescents Aged 9–17 Years: A Cross-Sectional Study in Guangzhou, China
Source: Nutrients. 2026 Jul 5;18(13):2188. doi: 10.3390/nu18132188 (PMC13363526; doi:10.3390/nu18132188)
Supplement: Supplementary file 1 [file nutrients-18-02188-s001.zip › nutrients-4360628-supplementary.pdf]

Figure S1. Flowchart of participant selection.

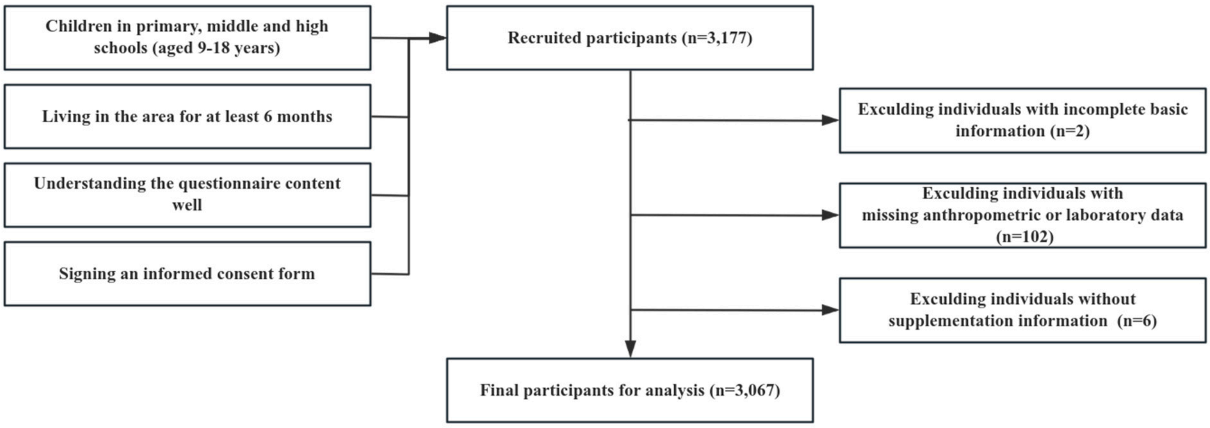

Table S1. Associations between serum 25(OH)D change and dyslipidemia outcomes.

**Binary Outcomes**	**Model 1**	**Model 2**	**Model 3**
	**OR(95%CI)**	**OR(95%CI)**	**OR(95%CI)**
Elevated TG	0.88 (0.77, 1.00)*	0.87 (0.75, 1.01)	0.88 (0.76, 1.02)
Elevated TC	1.19 (1.02, 1.37)*	1.24 (1.06, 1.46)**	1.22 (1.03, 1.43)*
Elevated LDL-C	1.21 (0.92, 1.58)	1.14 (0.83, 1.53)	1.15 (0.83, 1.55)
Reduced HDL-C	0.86 (0.75, 0.98)*	0.87 (0.75, 1.01)	0.86 (0.74, 1.00)
Elevated nonHDL-C	1.09 (0.93, 1.26)	1.09 (0.91, 1.28)	1.09 (0.91, 1.29)
Overall dyslipidemia	0.95 (0.87, 1.03)	0.96 (0.87, 1.05)	0.94 (0.85, 1.04)

Note: ORs represent the change in odds of the outcome associated with a one-SD increase in serum 25(OH)D levels.

*p<0.05, **p<0.01, ***p<0.001.

Table S2. The Threshold Effect of serum 25(OH)D levels on HDL-C.

**Outcomes**	**Effect (95%CI)**	***P***
Fitting model by standard linear regression	0.025 (0.014, 0.035)	<0.001***
Fitting model by two-piecewise linear regression		
Inflection point		
<14.42	0.076 (0.019, 0.133)	0.008**
≥14.42	0.015 (0.002, 0.029)	0.029*
P for likelihood test		0.039*

Note: *p<0.05, **p<0.01, ***p<0.001.

Effect estimate was β adjusted for the full model.

Table S3. Associations of serum 25(OH)D status and lipid profiles, stratified by ow/ob status.

**Lipid Profiles**	**VD Change/ Status**	**non-ow/ob**		**ow/ob**	
		**Adjusted β (95%)CI**	***P* for trend**	**Adjusted β (95%)CI**	***P* for trend**
TG	Per SD	-0.018 (-0.034, -0.001)*	/	-0.050 (-0.083, -0.017)**	/
	Deficiency	Reference		Reference	
	Inadequate	-0.012 (-0.059, 0.034)	0.087	-0.030 (-0.141, 0.081)	0.179
	Adequate	-0.038 (-0.090, 0.013)		-0.069 (-0.187, 0.049)	
TC	Per SD	0.076 (0.042, 0.110)***	/	-0.006 (-0.073, 0.060)	/
	Deficiency	Reference		Reference	
	Inadequate	0.128 (0.032, 0.223)**	<0.001***	0.089 (-0.133, 0.312)	0.684
	Adequate	0.209 (0.104, 0.314)***		0.080 (-0.156, 0.316)	
LDL-C	Per SD	0.031 (0.008, 0.054)**	/	-0.005 (-0.052, 0.042)	/
	Deficiency	Reference		Reference	
	Inadequate	0.068 (0.003, 0.133)*	0.022*	0.041 (-0.117, 0.198)	0.608
	Adequate	0.092 (0.020, 0.163)*		0.050 (-0.117, 0.217)	
HDL-C	Per SD	0.030 (0.017, 0.042)***	/	0.007 (-0.012, 0.027)	/
	Deficiency	Reference		Reference	
	Inadequate	0.068 (0.033, 0.103)***	<0.001***	0.022 (-0.044, 0.087)	0.829
	Adequate	0.096 (0.057, 0.135)***		0.017 (-0.053, 0.086)	
nonHDL-C	Per SD	0.046 (0.018, 0.074)**	/	-0.014 (-0.071, 0.044)	/
	Deficiency	Reference		Reference	
	Inadequate	0.060 (-0.019, 0.138)	0.008**	0.068 (-0.126, 0.261)	0.693
	Adequate	0.113 (0.026, 0.199)*		0.063 (-0.141, 0.268)	
TG/HDL-C	Per SD	-0.039 (-0.059, -0.020)***	/	-0.055 (-0.092, -0.018)**	/
	Deficiency	Reference		Reference	
	Inadequate	-0.062 (-0.116, -0.008)*	<0.001***	-0.046 (-0.171, 0.079)	0.199
	Adequate	-0.108 (-0.168, -0.049)***		-0.081 (-0.214, 0.051)	
TC/HDL-C	Per SD	-0.006 (-0.029, 0.016)	/	-0.022 (-0.069, 0.024)	/
	Deficiency	Reference		Reference	
	Inadequate	-0.052 (-0.116, 0.011)	0.298	0.023 (-0.132, 0.178)	0.808
	Adequate	-0.050 (-0.120, 0.020)		0.026 (-0.138, 0.190)	

Note: Adjusting for the full model except BMI z score. *p<0.05, **p<0.01, ***p<0.001.

Table S4. Interaction of serum 25(OH)D levels and ow/ob on dyslipidemia outcomes.

**Binary Outcomes**	**Adjusted OR (95% CI)**		$P_{interaction}$	**RERI(95% CI)**
	**non-ow/ob**	**ow/ob**		
Elevated TG	0.96 (0.79, 1.16)	0.75 (0.59, 0.96)*	0.413	0.01 (-1.01, 1.03)
Elevated TC	1.25 (1.03, 1.52)*	1.11 (0.80, 1.53)	0.434	0.06 (-1.99, 2.11)
Elevated LDL-C	1.21 (0.75, 1.93)	1.13 (0.75, 1.72)	0.872	0.58 (-6.31, 7.47)
Reduced HDL-C	0.75 (0.62, 0.91)**	1.14 (0.88, 1.49)	0.115#	0.41 (-0.58, 1.4)
Elevated nonHDL-C	1.24 (1.00, 1.53)	0.86 (0.63, 1.17)	0.174	-0.15 (-2, 1.71)
Overall dyslipidemia	0.94 (0.83, 1.06)	0.95 (0.79, 1.14)	0.653	0.22 (-0.51, 0.95)

Note: ORs represent the change in odds of dyslipidemia outcome associated with a one-SD increase in serum 25(OH)D levels, adjusting for the full model except BMI z score. *p<0.05, **p<0.01, ***p<0.001.

#$P_{interaction}$ was determined by the LRT test due to significant non-linear association between serum 25(OH)D level and reduced HDL-C (p=0.026).

RERI: relative excess risk due to interaction.

Table S5. Interaction of serum 25(OH)D level and ow/ob status on lipid profiles after adjusting the HDL-C.

**Lipid Profiles**	**Adjusted β (95%)CI**			$\mathbf{P_{interaction}}$
	**Total**	**non-ow/ob**	**ow/ob**	
TG	-0.024 (-0.039, -0.010)**	-0.016 (-0.033, 0.000)	-0.049 (-0.082, -0.016)**	0.367
TC	0.015 (-0.009, 0.0393	0.027 (0.000, 0.054)	-0.020 (-0.075, 0.035)	0.062
LDL-C	0.010 (-0.011, 0.0301	0.018 (-0.005, 0.041)	-0.010 (-0.055, 0.036)	0.382
nonHDL-C	0.015 (-0.009, 0.039)	0.027 (0.000, 0.054)	-0.020 (-0.075, 0.035)	0.062

Note: *p<0.05, **p<0.01, ***p<0.001. Total β was adjusted for the full model plus HDL-C, stratified β was adjusted for HDL-C plus the full model except BMI z score.

Table S6. Interaction of serum 25(OH)D level and ow/ob status on lipid profiles after excluding children and adolescents with thinness.

**Lipid Profiles**	**Adjusted β (95%)CI**			$P_{interaction}$
	**Total**	**non-ow/ob**	**ow/ob**	
TG	-0.025 (-0.040, -0.010)**	-0.016 (-0.033, 0.001)	-0.050 (-0.083, -0.017)**	0.375
TC	0.061 (0.030, 0.091)***	0.081 (0.047, 0.116)***	-0.006 (-0.073, 0.060)	0.013*
LDLC	0.025 (0.004, 0.046)*	0.036 (0.012, 0.060)**	-0.005 (-0.052, 0.042)	0.161
HDLC	0.025 (0.014, 0.036)***	0.031 (0.018, 0.043)***	0.007 (-0.012, 0.027)	0.156
nonHDLC	0.035 (0.010, 0.061)**	0.051 (0.022, 0.079)***	-0.013 (-0.071, 0.044)	0.019*
TG/HDLC	-0.043 (-0.061, -0.026)***	-0.039 (-0.058, -0.019)***	-0.055 (-0.092, -0.018)**	0.928
TC/HDLC	-0.009 (-0.029, 0.012)	-0.003 (-0.027, 0.020)	-0.022 (-0.068, 0.024)	0.246

Note: *p<0.05, **p<0.01, ***p<0.001. Non-linear association between serum 25(OH)D level and HDL-C was not significant (p=0.089).

Table S7. Interaction of serum 25(OH)D level and weight status on lipid profiles.

Lipid Profiles	Adjusted β (95%)CI			$P_{interaction}$
	**non-ow/ob**	**ow**	**ob**	
TG	-0.018 (-0.034, -0.001)*	-0.041 (-0.084, 0.002)	-0.052 (-0.109, 0.004)	0.444
TC	0.076 (0.042, 0.110)***	-0.014 (-0.104, 0.076)	0.015 (-0.089, 0.119)	0.031*
LDLC	0.031 (0.008, 0.054)**	-0.009 (-0.071, 0.053)	0.004 (-0.073, 0.080)	0.193
HDLC	0.030 (0.017, 0.042)***	0.005 (-0.022, 0.032)	0.010 (-0.021, 0.040)	0.297
nonHDLC	0.046 (0.018, 0.074)**	-0.019 (-0.097, 0.059)	0.005 (-0.085, 0.096)	0.045*
TG/HDLC	-0.039 (-0.059, -0.020)***	-0.046 (-0.095, 0.004)	-0.057 (-0.118, 0.004)	0.599
TC/HDLC	-0.006 (-0.029, 0.016)	-0.025 (-0.085, 0.035)	-0.005 (-0.084, 0.073)	0.455

Note: *p<0.05, **p<0.01, ***p<0.001.
